# Discussion on software aging management of nuclear power plant safety digital control system

**DOI:** 10.1186/s40064-016-3780-2

**Published:** 2016-12-12

**Authors:** Huihui Liang, Pengfei Gu, Jianzhong Tang, Weihua Chen, Feng Gao

**Affiliations:** State Key Laboratory of Nuclear Power Safety Monitoring Technology and Equipment, China Nuclear Power Design CO., LTD, Shenzhen, China

**Keywords:** Safety digital control system, Software aging factors, Software aging management

## Abstract

Managing the aging of digital control systems ensures that nuclear power plant systems are in adequate safety margins during their life cycles. Software is a core component in the execution of control logic and differs between digital and analog control systems. The hardware aging management for the digital control system is similar to that for the analog system, which has matured over decades of study. However, software aging management is still in the exploratory stage. Software aging evaluation is critical given the higher reliability and safety requirements of nuclear power plants. To ensure effective inputs for reliability assessment, this paper provides the required software aging information during the life cycle. Moreover, the software aging management scheme for safety digital control system is proposed on the basis of collected aging information.

## Background

The reactor status and power generation of a nuclear power plant are controlled by a digital control system. The digital control system can implement protective measures to maintain nuclear power plant safety in emergencies, as well as provides accurate information to the operator. The performance of the digital control system is directly related to the safety and economic operation of nuclear power plants. A variety of protective measures are taken by the digital system of a nuclear power plant. Software aging management enhances economic benefits and ensures the safe operation of nuclear power plants. Given that the aging management of the digital control system is a relatively new topic, no significant research on nuclear power plants has been established.

The aging management of nuclear power plant includes the following: (a) selecting safety systems, structures, and components; (b) understanding the aging process and identifying aging mechanisms; and (c) preventing and delaying the aging process. Aging management for digital control systems of nuclear power plants ensures that the systems are operating in an adequate safety margin throughout their life cycles (Simola 1999). IAEA NS-G-2.12 (2009) provides guidance for the aging management of nuclear power plants, as well as a reference for the aging management of critical systems, structures, and components. Researchers currently focus more on equipment aging management for nuclear power plants (John and Philippa 2006; Yang et al. 2012; Zeng et al. 2013). Software aging management is crucial because software is the core difference between digital and analog systems. Consequently, a perfect aging management program should be constructed and aging management measures should be implemented as early as possible for nuclear power plants.

The concept of software aging is that software performance decreases and the rates of crashes or undesired hang-ups increase after a long period of continuous operation (Thein et al. 2008). The typical causes of software degradation include memory bloating and leaking, unreleased file-locks, data corruption, storage space fragmentation, and accumulated round-off errors (Garg and Van Moorsel 1998). The master-slave serve switch failure occurs because of unreleased file-locks. A recent study showed that software aging exists in long-running digital control systems, which degrades system performance and causes hang-up failures. Computer system failures occur because of software factors instead of hardware failure (Gray and Siewiorek 1991). The key steps in aging management are the evaluation and prediction of software aging. A paper discussing the synergy between nuclear security and safety has provided an evaluation methodology for nuclear system and a reference for software aging assessment (Cipollaro and Lomonaco 2016).

The aging management of digital control system software for nuclear power plants is analyzed on the basis of the characteristics of the safety digital control system. This paper is structured as follows: section one introduces the background. Section two analyzes the characteristics of software for the digital control systems of nuclear power plants. Section three provides information on software aging management, which should be collected during system lifetime to provide effective inputs for managing software aging. Section four proposes a schema for software aging management. Section five presents the conclusions.

## The characteristics of safety digital control system

In contrast with the analog system, the digital control system can overcome physical limitations in hardware by the introduction of a software system, as well as handle complex logic and calculation functions. This fail-safe and fault-tolerant technology can be conveniently and effectively executed. The safety digital control system of nuclear power plants possesses a considerable amount of digital information. It can effectively perform power station real-time state supervision, diagnostic, calibration and performance assessment.

The design principles of the digital control system include single failure criterion, independence, common cause failure criteria, and capability for testing and calibration. In-depth defensive features and defense should be designed for the safety software system. To prevent software instability, limit checks, logic check for error input date, and assigned default values should also be included. Therefore, the digital control system software is vast and complex. The source codes of the digital control system can reach hundreds of thousands lines. However, design flaws or insufficient requirements, which may result in software failures, are unavoidable during software development. Failures of the digital system software are difficult to intuitively check, classify, and correct, which may affect performance. In addition, influence (e.g. the compatibility) is difficult to evaluate during software revisions and updates.

**Fig. 1:**
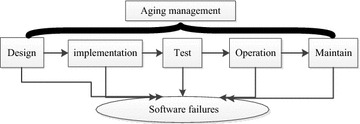
Life cycle of software

The software aging failure mechanism is completely different from that of hardware. Hardware failures can be found or resolved by online software monitoring, diagnosis, correction, hardware redundancy, and other methods. Software failures do not occur at random, unlike hardware failures. Software errors and defects cannot be completely discovered through exhaustive testing. Software aging management for the digital control system is necessary to ensure the long-term, reliable, and safe operation of nuclear power plants. Based on IEEE standard 7-4.3.2 (2010), a computer system consists of the following life phases: conceptual design, detailed design, implementation, testing, installment, site acceptance testing, operation, maintenance, and retirement. As shown in Fig. 1, this paper divides the software life cycle into design, implementation, test, operation and maintenance. Different types of software failures may arise from any software life phase. Software aging management should be executed from the design phase to fully gather information on software aging.

## Software aging management information

Hardware aging management for nuclear power plants has been verified for decades, and its relevant regulations and standards have also matured. Software aging management is a core, but difficult, point of the digital control system. Testing and review technology can reduce software defects, but cannot guarantee that the software system is faultless.

Hardware often requires repair or replacement when failure occurs; then, associated software should be redesigned or upgraded. Redesigning and upgrading may introduce new design defects, which may cause new failures. Information for software aging management should be collected throughout the phases of software design and maintenance. Both quantitative and qualitative data should be recorded and stored to ensure software lifetime, which will provide the input for predicting and evaluating software aging. A reliable software aging management strategy should be designed in combination with the recorded aging information. The relevant information for using in the software management process is listed on Table 1. Safety and security design are necessary for the digital control system and are the first safeguard against software aging management. In the implementation phase, the developmental methods and instruments are related to software fault style and aging mechanism (e.g., the different compile language corresponds to the different failure risk). Software weakness can be identified by verification and validation technology in the implementation phase. Subsequently, to evaluate the aging phenomenon, users can use information rooted in software verification and validation. Software aging information mainly comes from the system and human feedback in the operation and maintenance phase.

**Table 1:** Software aging management information

Phase	Software aging management related information	Collection method
Design	Performance requirements (precision, response time, etc.)	Review and analysis
	Fault-avoidance technology	
	Self-diagnosis technology	
	Self-supervision technology	
	Safety and security technology	
	System stability and reliability outside factors, e.g. environment	
Implementation	Design deviation	Traceability analysis
	Software configuration	Feedback and evaluation
	Assumptions and boundary	
	Data and structure specification	
	Input and output specification	
	Weak spot of the software development	
Test	Large date volume test	Software validation and verification
	Carrying capacity	Testing
	Concurrency capacity	
	Fatigue strength	
	Fault-tolerant ability	
	Network quality (clock recovery, network timing and packet loss, etc.)	
	Abnormal problems	
Operation	Accumulation of round-off errors	Feedback and statistics
	Aging-related bugs	Evaluation
	Resource exhaustion, e.g. CPU, disk memory and network, etc	
	Unplanned failures	
Maintain	Maintain records	Power plant feedback
	Upgrade records	Evaluation

## Software aging management scheme

**Fig. 2:**
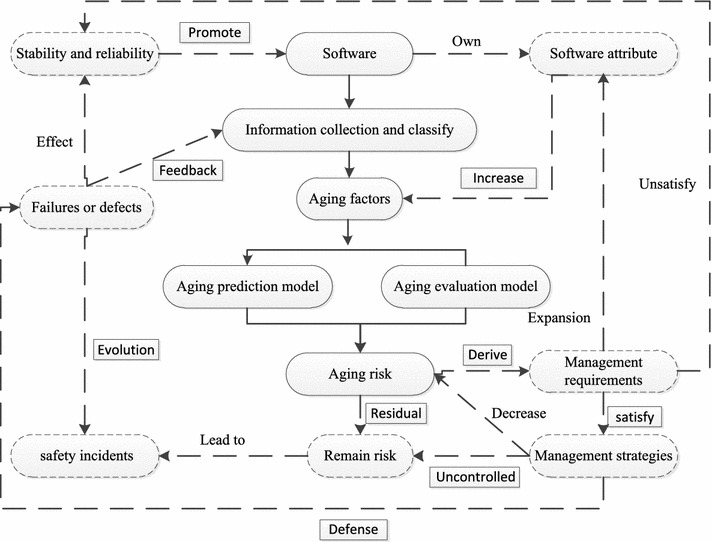
Software aging management schema for nuclear power plant safety digital control system

Figure 2 shows the software aging management scheme combined with software aging information that is collected throughout the software design and maintenance phases. In Fig. 2, the solid lines represent the software aging analysis process and the dotted lines represent the outcomes and ease measures.

1. Aging process
  Information collection and classification
    The information that includes failures and defects should be classified as time-based or requirement-based. Then, the qualitative and quantitative aging factors are obtained.
  Prediction and evaluation model for aging.
    The method of aging prediction and evaluation can select statistical techniques, machine learning, and Markov decision.
  Aging risk.
    Aging risks will be exposed through the above aging prediction and evaluation model. Then, aging management strategies will be designed.
2. The steps of software aging management
  Aging risks are derived to update aging management requirements.
  Aging risks will decrease when aging management requirements are satisfied by aging management strategies. However, aging risks will affect the stability and reliability of the software if these requirements are unsatisfied. Therefore, aging management strategies can defend against software failures or design defects.
  Software failures or design defects affect software stability and reliability, and may result in safety incidents. Therefore, failures and defects can be avoided by providing sufficient aging information.
  Residual aging risks are uncontrollable by aging management strategies and may lead to safety incidents. To improve the stability and reliability of the safety digital control system, software attributes become more complex. Although stability and reliability goals promote software development, these factors increase software aging factors.

Aging management of digital control system software for nuclear power plants improves the software aging process by the following measures:

1. Design phase
  Fault-avoidance technology,
  Fault-tolerant technology,
  Self-detection and self-diagnosis,
  Safety and security design.
2. Implementation and test phase
  Reliability as the core quality objective,
  Determining a quality metric (requirements analysis, design, testing, and acceptance) of every phase,
  Analyzing the risks and consequences caused by common software failures,
  Software validation and verification.
3. Operation phase
  Executing online tests by combining reliability operations with maintenance,
  Relevant and documented operational feedback for support software,
  Monitoring and feedback of software system performance parameters.
4. Maintenance phase
  Periodic testing and maintenance for software,
  Running maintenance of hardware and software,
  Assessing software upgrades.

## Conclusions

Researchers should focus on the aging management of the structures, components, and systems of the nuclear power plant digital control system. Aging management of the digital control system software is a weakness. This paper analyzes the aging mechanism and process of the safety digital control system software. Then, software aging information is collected throughout the design to maintenance phases. This paper describes the key aging points in different life phases. Finally, to improve the reliability of digital control systems, the aging management schema is proposed, which includes the aging process and management measures. Future research should focus on rejuvenation strategies for aging safety digital control systems of nuclear power plants.
